# A qualitative study on expert perspective for implementing and utilizing non-communicable diseases research findings

**DOI:** 10.1038/s41598-025-23036-5

**Published:** 2025-11-10

**Authors:** Mojgan Zareivenovel, Leila Nemati-Anaraki, Shadi Asadzandi, Bahram Rasoulian

**Affiliations:** 1 Department of Medical Library & Information Science, School of Health Management & Information Sciences, Iran University of Medical Sciences, Tehran, Iran; 2 Health Management and Economics Research Center, Health Management Research Institute, Iran University of Medical Sciences, Tehran, Iran; 3 Neurophysiology Research Center, Institute of Neuroscience and Cognition and Department of Physiology, School of Medicine, Shahid Beheshti University of Medical Sciences, Tehran, Iran

**Keywords:** Implementation, Research utilization, Knowledge implementation, Evidence-based, Implementation science, Non-communicable diseases, Problem-oriented, Health care, Health humanities

## Abstract

Today, all important and influential decisions must be based on the research finding. Research serves as a pioneer and foundation for precise and targeted planning and decision-making. The acceleration of applying and implementing health research finding, particularly in non-communicable diseases (NCDs), is crucial as it relates to the promotion of public health and improving the health and well-being of the population. This requires attention to a knowledge and evidence-based decision-making ecosystem. The present study aims to identify the perspectives of decision-makers in Iran’s research system regarding prerequisites, barriers and facilitators affecting the implementation of research findings in the field of non-communicable diseases. This qualitative study employed a purposive sampling method with 21 individuals who met predefined criteria. Interviews were conducted until data saturation was achieved. The interviews were conducted virtually and in person with deputy head of research and technology in medical sciences universities, managers of research development and evaluation, directors of research centers, head of medical information management and scientific resources centers, policymakers, researchers, and experts in knowledge implementation in the NCDs field. Thematic analysis and open coding with the MAXQDA software was employed for data analysis. Subsequently, a survey form was used to gather expert opinions on refining the coding process. Of the participants, 62% were male and 38% were female. Additionally, 52% were deputy head of research and technology, research managers, and directors of research centers, while 38% were policymakers and a few were researchers in NCDs field. The thematic analysis of the 21 interviews resulted in 591 codes, which were ultimately categorized into eight main categories and 56 subcategories. Two categories: “Scientific leadership (Research Railing)”, and “Strengthening and developing responsive policies” were identified as basic requirements, Four categories: “Designing, updating and upgrading health information systems”, “Designing, compiling and presenting organizational mechanisms”, “Information facilitation”, and “Provision financial resource and providing an economic platform” were identified as facilitators, while two categories: “Political Barriers” and “Organizational/Trans-organizational challenges” were identified as challenges impacting the implementation of research finding. The study’s findings indicate that to implement research finding, overarching policies within the country’s research system require Serious action. However, there are challenges in the implementation process that, if ignored, could pose threats. Each identified influencing factor in this study has independent potential to impact. Health system managers and policymakers in the NCDs field can leverage the findings of this study- which align with the expectations of research and technology vice presidents and managers at universities- in their future planning and policymaking related to strengthening a problem-oriented research approach and utilizing implementation science.

## Introduction

In today’s rapidly evolving world, all significant and impactful decisions should derive from valid research finding and closely aligned with the actual needs of the population. As emphasized in the 2004 World Health Organization (WHO) report titled “Knowledge for Better Health”, the real value of research lies in its ability to enhance public health when its outcomes are actively reflected and applied across various sectors^1^. The latent potential of research is multifaceted, serving not only as a strategic framework but also as a powerful tool for decision-making across a wide spectrum of stakeholders, all of whom rely on research to inform their actions and policies, including policymakers, government officials, healthcare system executives, NGOs, international organizations, and service providers^2^. The reality is that research conducted in various countries has the potential to address issues faced by healthcare providers, if there were based on their needs and effectively implemented at the right time^3–7^. Historically, communicable diseases were seen as the greatest health challenge worldwide, leading to a significant focus on their control and prevention. However, over the past few decades, substantial advancements in health systems and technologies have significantly improved the prevention and treatment of communicable diseases. In contrast, due to lifestyle changes (e.g., consumption of processed foods, decreased physical activity, and high-fat diets), Non -communicable diseases have increasingly emerged as major public health issues^8,9^. Currently, Non -communicable diseases disproportionately affect low- and middle-income countries, accounting for nearly one-third of deaths in these regions^10^. The WHO reported a 15% increase in mortality from Non-communicable diseases from 2010 to 2020 largely due to insufficient intervention and prevention strategies^11^.

This is highlighting the necessity of not confining knowledge within academic institutions and merely satisfying ourselves with publishing articles in specific journals. The mere act of publishing research finding in specialized journals is insufficient. Instead, there must be a concerted effort to accelerating the implementation of research finding in non-communicable diseases, as it directly relates to enhancing public health and improving population well-being. This demands a necessitating attention to a knowledge and evidence-based decision-making system. Despite the growing emphasis on evidence-based medicine, studies have shown that less than 20% of research finding are translated into clinical practice, thereby limiting their potential to improve patient outcomes. This glaring disconnect between research and practice highlights the urgent need for reform in research policy and the implementation process to ensure that the goals of improved health outcomes are realized^12^. In recent years, the scientific community has increasingly focused on the practical implications and effective application of generated knowledge in healthcare systems. But, to have a tangible impact on patient care, it is essential to foster a mutual understanding between researchers, knowledge disseminators, policymakers, and healthcare managers. Without this collaboration, research finding being confined to the pages of academic journals, offering little benefit to patients or healthcare systems. In the context of Iran, a developing country with limited resources, the need to bridge the gap between research and decision-making is even more pressing. Establishing a logical, evidence-based connection between research outcomes and the policymaking process is vital to ensure that scarce resources are used effectively^13^. This study aims to identify the key factors affected to the implementation of research findings in the field of NCDs; by doing so, it will provide critical insights that can enlighten future health policies and decision-making processes. The outcomes of this research have the potential to address current gaps in the management of NCDs, ultimately contributing to better health outcomes for the population and more effective utilization of research in practice.

## Methods

### Ethical consideration

This study was approved by the Ethics Committee of Iran University of Medical Sciences under the ethics code: IR.IUMS.REC.1400.1152. All the steps/methods were performed following the relevant guidelines and regulations. Informed consent was obtained from all participants.

### Participants

To determine the dimensions and components of implementing research findings in the field of non-communicable diseases, interviews were conducted with deputy head of research and technology in medical sciences universities, managers of research development and evaluation, directors of research centers, head of medical information management and scientific resources centers, policymakers, researchers, and experts in knowledge implementation in the field of non-communicable diseases. Selection criteria for participation included individuals who had one or more than one of the following: at least 10 years of executive experience in non-communicable diseases policymaking or decision-making; at least 5 years of executive or managerial responsibility in knowledge implementation or management of non-communicable disease research centers; or at least 10 years of research experience in non-communicable diseases field. Interviews were conducted using purposive sampling^14^. We followed a multi-stage process to identify and select eligible participants. First, we compiled a list of academic faculty members using the Iranian Scientometrics Information Database (ISID). Second, we prepared a list of (NCDs) research centers. Third, we identified the deputy heads of research and technology and head of medical information management and scientific resources centers. In addition, a separate list of ministerial policymakers and researchers was gathered. All lists were subsequently reviewed according to the predefined inclusion criteria. Interviews were conducted until data saturation^15^. This means that the researcher encountered data that was not new and was repeated frequently. To confirm theoretical saturation, two additional interviews were conducted. Ultimately, interviews were conducted with 21 individuals. The interviews due to the limitations as the special circumstances of the world that was affected by the coronavirus were conducted in three formats: in-person, via phone call, and online, using platforms like Skype, Google Meet, and other social media. In total, five in-person interviews, seven phone call interviews, and nine online interviews were conducted.

### Data collection

During this stage, an interview guide was used to gather data. This guide was developed through a scoping review which included an introduction and two main sections. The introduction presents the research title and objectives for the Interviewees. The other two sections consisted of open-ended questions: the first section gathered demographic information, such as academic rank, current job position, and executive and managerial roles. The second section addressed discussions related to implementing research finding and explored influencing factors and existing challenges. At the end of the interview, two general questions were posed to obtain supplementary opinions and suggestions from the interviewees. The first interview served as a guide to review the number of questions, interview duration, and finalize the interview guide. Each interview was allocated a time frame of 30 to 90 min. For data collection during the interviews, audio recording app was used after obtaining permission from the interviewees. After each interview, the recorded content was reviewed, and the interview notes were transcribed using Microsoft Word. To ensure data confidentiality, samples were coded.

### Data analysis

After each interview, transcription was immediately carried out using Microsoft Word. The transcription files were then imported into MAXQDA software for content analysis. Thematic analysis and open coding were utilized for data analysis. In the open coding approach, concepts were labeled, and categories were defined and developed based on their characteristics and dimensions. To gain a deep understanding of the interview texts for accurate data analysis, the researcher listened to each recorded interview multiple times to extract the main themes and key issues. Initial codes were determined based on this process, and sub-codes were categorized under each main code according to thematic and semantic similarities. The codes were repeatedly compared and classified into related categories. The same process was repeated for subcategories. During the coding and categorization, meetings were held with members of the research team to review and revise the extracted codes. All interviews were coded in the same way. After the initial analysis and coding, the codes were reviewed for validity by the supervisor and research advisors. Participants were also asked to review the codes and feedback was obtained^16,17^. Ultimately, both main and subcategories were identified. Descriptive information about the study participants is provided in Table 1.

**Table 1:** Demographic information of the interview participants.

Number	Gender	Educational Degree	Field of Study	Organizational Specialty	Interview Method
p1	Male	PhD^a^	Epidemiology	NCDs^b^/Policy-making	In-person
p2	Male	PhD	Epidemiology	NCDs/Policy-making	Online (Google Meet)
p3	Male	PhD	Biomedical Sciences	NCDs/Policy-making	Phone
p4	Female	PhD	Epidemiology	NCDs/Policy-making	In-person
p5	Male	MD^c^, PhD	Physiology	NCDs	In-person
p6	Male	PhD	Neuroscience	NCDs	In-person
p7	Female	PhD	Nutrition Sciences	NCDs	Online (Google Meet)
p8	Male	MD	Medicine	NCDs	Telegram Messenger
p9	Male	PhD	Library & Medical Information Science	Knowledge Implementation/Knowledge Management	Online (Google Meet)
p10	Male	PhD	Physiology	NCDs	Online (Skype)
p11	Male	PhD	Health Policy	Policy-making	Online (Google Meet)
p12	Male	PhD	Health Information Management	Policy-making	Online (Google Meet)
p13	Female	PhD	Health Policy	Policy-making	Bale Messenger
p14	Male	PhD	Physiology	Research Management/Evidence-based Medicine	Phone
p15	Female	PhD	Epidemiology	NCDs	Phone
p16	Male	PhD	Library & Information Science	Knowledge Implementation/Knowledge Management	Phone
p17	Male	PhD	Information Science	Knowledge Implementation/Knowledge Management	Phone
p18	Female	PhD	Clinical Psychology	NCDs	In-person
p19	Female	PhD	Library & Medical Information Science	Knowledge Translation	Online (Google Meet)
p20	Female	MSc^d^	Health Education & Promotion	NCDs	Phone
p21	Female	PhD	Library & Medical Information Science	Knowledge Translation	Phone

a: PhD (Doctor of Philosophy); b: NCDs (Non-Communicable Diseases); c: MD (Doctor of Medicine); d: MSc (Master of Science).

## Results

Of the participants, 62% were male and 38% were female. Among them, 52% were deputy head of research and technology in medical sciences universities, managers of research development and evaluation, and directors of research centers, while 38% were head of medical information management and scientific resources centers, policymakers and researchers in the field of non-communicable diseases. Using an open coding approach, 591 codes were initially identified. After eliminating duplicates and merging similar codes, in final stage, codes were classified into 8 main categories and 56 subcategories, representing the influencing factors on the implementation of research finding as requirements, facilitators and challenges. According to finding, prerequisites were categorized into two main areas: “Scientific leadership (Research Railing)”, and “Strengthening and developing responsive policies” with a total of 15 key concepts; effective facilitators for implementing research results were categorized into four main areas: " Designing, updating and upgrading health information systems,” “Designing, compiling and presenting organizational mechanisms”, “Information facilitation,” and “Provision financial resource and providing an economic platform,” with a total of 16 key concepts, while “Political Barriers” and “Organizational/Trans-organizational challenges” included 25 key concepts as influencing factors on the implementation of non-communicable disease research finding. Detailed explanations to gain a comprehensive understanding were provided in Tables 2, 3 and 4.

**Table 2:** Requirements influencing implementation and utilization of Non-Communicable Disease (NCD) research findings (Prerequisites).

	Theme	Sub-theme
Requirements	Scientific Leadership	Guiding the general policies of the research system
		Streamlining and moving towards academic scientific discourse
		Changing Evaluation and promotion policies for faculty members
		Decentralization of research priorities in accordance with climatic and regional conditions
		Providing an appropriate mechanism for appointing health deputy ministers
		Providing a mechanism for employment research experts in health centers
		Designing and compiling short-term educational courses on implementing science
		Providing promotional and culture-building policies for the credibility of science
	Strengthening and Developing Responsive Policies	Guiding and strengthening the participation of faculty members and students
		Creating and strengthening p2p* networking for implementing the finding of non-communicable disease research
		Designing and planning packages based on modifying people’s lifestyles through preventive approach
		Strengthening communication between policymakers and academics
		Strengthening and providing existing infrastructure for designing integrated systems
		Designing tools for measuring effectiveness
		Setting up common realization committees

* Peer-to-peer networking.

**Table 3:** Facilitators influencing implementation and utilization of Non-Communicable Disease (NCD) research findings.

	Theme	Sub-theme
Facilitators	Designing, updating and upgrading health information systems	Updating and promoting data storage existing systems
		Strengthening and updating research proposal information system for various research, educational, and health purposes.
		Designing a national, regional, and international system to access evidence
	Designing, compiling and presenting organizational mechanisms	Developing frameworks, drafting regulations, and enacting effective laws
		Simplifying processes and reducing administrative bureaucracy
		Expanding clinical knowledge management units, libraries, and scientific resource management to facilitate the implementation of NCD research finding
		Establishing legal and regulatory supportive mechanisms for evidence-based decision-making
		Updating, monitoring and systematic identification of research priorities and needs
		Activating Health system Research (HSR)
		Involving representatives from healthcare units and NCDs centers in research councils
		Monitoring and evaluating research evidence at specified intervals
	Information Facilitation	Setting up think tank room and monitoring research needs and foresight
		Forming team science with diverse expertise
		Critiquing evidence-based managerial decisions
	Provision financial resource and providing an economic platform	Targeting research budgets, improving financial support for researchers, and involving researchers in the benefits derived from research projects
		Securing funding sources, ensuring continuity of financial and moral support

**Table 4:** Barriers influencing implementation and utilization of NCD research Findings.

	Theme	Sub-theme
Barriers	Political Barriers	Government-imposed policies
		Conflicting political interests with research finding
		Personal political motivations
		Limited access to information
		Lack of cooperation or willingness of the policy maker in implementation
	Organizational/Trans-organizational challenges	Lack of a clear roadmap for research implementation
		Island approach in research systems
		Disconnect between university deputies
		Distance between policymaker and research
		Focus on treatment-oriented rather than prevention -based research
		Contradictions between managerial experiences and research finding
		Research managers opinion in policy-making
		Distance between the curriculums and educational needs with conditions of the society
		Weak dissemination of national project result
		Failure to separate academic needs from clinical needs
		Conducting research according to the research tendency of journals (problematization instead of problem-finding)
		Failure to identify academic capacities and weakness in research customization
		Unbalanced distribution of specialists in recruitment and employment
		Weak communication between groups and specialties out of organization
		Paperdemic*
		Undefined access levels
		Lack of management stability among managers and policymakers
		Weak and supportive morale at the individual and organizational level
		Financial concerns for providing research needs
		Relationalism in ordination

* Paperdemic: The phenomenon of the outbreak of publishing enormous numbers of articles placing a conspicuous burden on journals and yet having little scientific significance.

### Effective requirements for implementing research findings in non-communicable diseases (Prerequisites)

The requirements for implementing research finding are placed at fundamental level. Scientific leadership and strengthening responsive policies are the foundation parameters for implementing research finding. Issues such as providing orientation to the Iran’s research system, changing evaluation system, and strengthening communication between policymakers and researchers are among the main issues that need to be considered.

### Theme 1- scientific leadership

Research is one of the key components of the scientific system and is the foundation for the development of new technologies, the commercialization of scientific achievements, and the utilization of the capacity of knowledge to improve human life. Research in the field of non-communicable diseases is one of the main issues in the country. For this reason, all countries are trying to develop research in their countries, remove barriers and problems, and provide the basis for deepening research in all aspects of the country. Studies show that there are no clear and specific policies in the Iranian research system regarding the implementation of research findings, especially research in the field of non-communicable diseases. So, before any action, a suitable base must be provided.

*“I still believe*,* in our country*,* we do not have a stable and consistent policy. Unfortunately*,* the lack of consistent policies has led to decisions being made based on a situation” (p14)*.

*“The approach of truly defining research based on the country’s needs has not yet taken hold. It may have improved in recent years*,* but I think the main goal is still to publish articles rather than solve the country’s problems. Naturally*,* publishing articles is within the boundaries of knowledge and our problems does not necessarily correspond to the boundaries of knowledge” (p5)*.

### Theme 2- strengthening and developing responsive policies

In the last decade, governance and development thinking have been given proper attention. Paying serious attention to development policies means paying attention to accountability mechanisms and the legitimacy of processes because policies are reflected in everyday social interactions^18^.

*“We must be educated not to elect a president or a representative based on our personal interests. The public must learn to vote for those who make decisions in the public interest based on scientific finding*,* and strengthen this relationship between politicians and academics.“(p11)*.

### Effective facilitators for implementing research findings in Non-Communicable diseases

The category of effective facilitators indicates how much existing infrastructure and technologies in the country have been utilized and how much can still be leveraged. The effective facilitators identified include “Designing, updating and upgrading health information systems”, “Designing, compiling and presenting organizational mechanisms”, “Information facilitation”, and “Provision financial resource and providing an economic platform” which will be elaborated below.

### Theme 1- Designing, updating and upgrading health information systems

According to the interviewees, designing systems for policymakers to utilize research finding is essential, particularly those that relate to national, regional, and global issues. Many systems exist within the Ministry of Health and Medical Education, if upgraded with necessary infrastructures, could facilitate the use of research findings for policy-making in the realm of non-communicable diseases. Interviewees suggested Strengthen and upgrade the Research Proposal Information System (RPIS) in which to include reasons for the success and failure of undertaken proposal.

*“Cochrane has a slogan that says “Trusted evidence. Informed decisions. Better health “(p.14)*.

### Theme 2- Designing, compiling and presenting organizational mechanisms

Participants emphasized that for the implementation of non-communicable disease research finding to becoming an organizational habit, regulations need to be established. Organizational bureaucracies were cited as one of the main barriers impeding progress and discouraging researchers. Simplifying processes could transform these barriers into facilitators. Most interviewees noted that utilizing the capacities of clinical knowledge management and scientific information management units could help to create a conducive environment for implementing research finding and prevent the establishment of new structures. Furthermore, supporting individuals would improve their performance to foster a research-based decision-making culture. Another factor influencing the prevention of non-communicable diseases and the best effect of researches is the activation of Health System Research (HSR). The participants in the interview admitted that in order to be aware of the research needs in the field of non-communicable diseases in universities, it is better to have representatives from the treatment deputy in Health System Research.

*“A legal mechanism or either legal or organizational is needed. It means that I am required to do this work*,* just as I am now promoted with an article*,* it should come into the structure. If you want to be promoted*,* do such. You must have told someone that who is a decision maker or a policy maker is required to follow my data” (p.9)*.

### Theme 3- information facilitation

One of the suggestions raised by interviewees was to establish think tanks rooms aimed at providing actionable solutions to problems, fostering a culture of participation among experts, and offering intellectual and consultative supports to policymakers. It was also recommended to invite specialists for better identification and aggregation of research needs. For evidence-based decision-making facilitation, it was proposed that assistants be appointed alongside managers.

### Theme 4- provision financial resource and providing an economic platform

Interviewees indicated that supportive financial systems for research require a knowledgeable vision. To secure funding and address financial support shortcomings, collaborative budgets should be defined, and utilizing external sources, grants, and specialized scholarships. They also noted that legal, and procedural guarantees for supporting researchers would enhance sustainability and productivity. Researchers must feel their contributions are valued, and all necessary tools and resources should be provided to prioritize research objectives. Participants suggested that sharing benefits from research with researchers would increase their sense of responsibility for the projects. Overall, the finding highlights that effective facilitation in implementing research finding in non-communicable diseases relies heavily on existing infrastructures and the establishment of supportive systems.

*“The costs of health care are increasing every day in the world*,* and in developed countries*,* their research budgets are proportional to these increasing costs.” (p.14)*.

### Challenges in implementing research findings in Non-Communicable diseases

Based on qualitative analyses, political barriers and organizational/trans-organizational challenges significantly hinder the implementation of research finding in the field of non-communicable diseases. It seems that public policy making in Iran is sometimes very politicized, hasty and temporary. It should be noted that in countries like Iran, science has a special dependence on politics, because the politicians and managers must formulate and implement the development plan. Additionally, various gaps and inadequacies in existing laws and regulations have resulted in inefficiencies within research-driven organizations. In this context, it is necessary to adopt a comprehensive and integrated management approach by reforming laws and the integration of overlapping entities to transform existing threats into opportunities.

### Theme 1- political barriers

Interviewees pointed out that the research system serves as a practical arm for national development, yet political currents often overshadow scientific processes. The intervention of political actors can impede evidence-based decision-making, as decisions frequently prioritize public political interests. Such politicization can manifest at both individual and structural levels, leading to bias in decision. Furthermore, sanctions have restricted access to global databases, complicating information retrieval for researchers.

*“Our country is highly politicized. Representatives interfere in this work. Well*,* managers never make decisions based on evidence” (p11)*.

*“Many times*,* we see that the public policies of the country also affect these issues*,* that is*,* due to political discussions*,* social*,* and financial issues*,* the results of our researches may not be included in the policy making system” (p8)*.

### Theme 2- Organizational/Trans-organizational challenges

Participants highlighted the lack of a clear roadmap for achieving research goals, resulting in confusion and unrecognized priorities. On the other hand, Ineffective information systems in health care and poor communication between researchers and data sources have led to data congestion. The weak cooperation and interaction of health centers with university deputies in providing information and awareness of the problems of the centers is another major challenge. According to the interviewees, the separation of university deputies, including treatment, education, research and technology, and even health deputies, has made them unaware of their deputy research priorities. The absence of a problem-oriented approach in non-communicable disease research field may lead to negative outcomes, because most studies focus on treatment rather than prevention. Participants expressed frustration with the accessibility of research finding. Based on their opinion, managers, policymakers, researchers, and specialists often don’t have adequate access. Furthermore, the absence of customization research approach in universities wastes both tangible and intangible resources. Also, repetitive and unnecessary projects, in different educational levels, has led to dilute research relevance and applicability. Sometimes the researches are not aimed at solving the problems of the society, in contrast, they are focusing on the desire of prestigious journals. In fact, researchers seek to meet the needs of journals and policy makers of other countries. Regarding the present research, understanding societal challenges such as diabetes, obesity, and lifestyle factors, as well as prevention methods tailored to the community, is highly beneficial. Several interviewees expressed that the lack of a cohesive structure for disseminating findings—limited primarily to reports and administrative communications—hinders timely implementation. Another factor was weakness of teamworking and a lack of supportive culture among managers and researchers at the individual and organizational level. Finally, financial shortages in research and technology deputies exacerbate the challenges faced by researchers and faculty members. Overall, a strategic focus on prevention, along with enhanced cooperation and resource allocation, is vital for effectively addressing non-communicable diseases.

*“We mainly do not have demand-oriented research. We have a gap in the problem and its identification. In healthcare*,* the situation is getting worse. We should consider prevention more than treatment.” (P.19)*.

*“We don’t have a road map at all to say that we are going to implement the research finding*,* then how do we expect this concept of implementation to be accepted in the universities” (p. 2)*.

## Discussion

This study aimed to identify the dimensions and components influencing the implementation of research findings in the field of non-communicable diseases, based on the perspectives of decision-makers and policymakers within the research system at medical universities in Iran. The interviewees outlined various prerequisites, challenges and facilitators that could significantly affect the implementation of research finding. Based on the coding of interviews, 56 subcategories were identified, organized into eight main categories under the themes of prerequisites, barriers and facilitators. The qualitative analysis indicated that implementing research finding requires enhancement and strengthening. Despite extensive research efforts, the knowledge implementation remains challenging. Identifying these factors can greatly aid in understanding and implementing the research finding effectively.

This study revealed that Iran’s research system needs to change and reform the policies and evaluation indicators of universities and faculty members; Also, the thesis-oriented research approach should be reformed and must solve the problems of society; In addition, flexible incentive and support policies for researchers should be prioritized. According to the dynamic essence of science, it must be integrated into practical life. Therefore, research system policies should move from the approach of merely publishing research results in journals to focus on their application and implementation. In this regard, studies consistent with the results of the present study, have highlighted that the evaluation indicators been selected appropriately, and a gap has been created between the scientific community and their activities^19,20^. Effective strategies should be used to have greater productivity of human resources. According to the interviewees, decision -makers and policymakers in the research system must come to the common understanding that acting in the field of research and the implementation of its findings requires stability and coherence. Actually, long-term planning can play an important role in making research capacities more efficient.

Another issue is, the existence of multiple research systems which focused merely on storing data, it can be leveraged effectively to address existing problems by utilizing this stored information. Supporting this, Leon et al., emphasized that an effective health information system can provide essential data for health system management, accountability, planning, policymaking, monitoring, and quality improvement^21,22^. One significant challenge in health policymaking is the reliance on overly simplistic methods for decision-making, which can lead to stakeholder disagreement and weak policy execution. If decisions are incorrect, adverse side effects may ensue. Studies affirm that the research system must align with the principles related to an effective health policymaking cycle. In this regard, Damari et al., outlined seven characteristics of a health policymaking system, including stakeholder engagement, social accountability, enforceability, meritocracy, stable stewardship, reliance on evidence, and collaboration and coordination capabilities^22^. On the other hand, to effectively implement research finding in the non-communicable disease domain, special legislative support is essential because the global landscape rapidly changing, laws must be updated, and monitoring compliance with these laws is equally critical. Aligning with the findings, Markazmalmiri asserts that law is a primary governance tool, ensuring not only the establishment of institutions but also the enhancement of efficiency and the guarantee of citizens’ rights^23^. Transformations in systems and methodologies within the research framework are imperative for enhancing service quality and expediting processes. Redesigning the missions and functions within the Ministry of Health and Medical Education will lead to significant transformations. Research by Hosseini et al., one of the barriers in the relationship between university and industry in Iran, is the lack of sufficient communication between university research, especially academic theses and dissertations, with the needs of industry^24^. Malekafzali et al., also highlighted that no health system can provide all necessary services without prioritizing specific issues. Therefore, prioritization is one of the most important issues in any health care system^25^. The most basic action is determining research priorities as the key and starting point of the research management cycle. Ebrahimi and et al., suggested regular meetings involving representatives from various health deputies to identify and prioritize research needs as a viable strategy for overcoming evidence-based policymaking barriers^26^.

Given that policy of science, is shapes our world, policymakers often have different interest with researchers. When there is a willingness for change or policy reform, these groups can provide substantial moral and practical support^27^. Seyedian emphasized that the negative and challenging consequences of politicization among societies, especially in Iran, is one of the most important topics has been the focus of the analysts in recent years^28^. The finding indicates that the intervention of political actors and institutions in specific situations makes decisions based on political interests instead of research finding. In other words, the interests of the government should be aligned with the general interests of public. Moghtadaie and Azghandi argue that a government is considered legitimate when the values ​​and thoughts of the public do not conflict with the values ​​and thoughts of the elites of the society. If a society experiences a conflict between national, public, and the government interests, opportunities for development will diminish^29^. In this regard, Soori pointed out three persistent issues: the making of national decisions without using scientific finding, inadequacies of locally derived scientific findings, and the lack of evaluation of interventions during epidemics. The failure to utilize the capacities of universities and research centers hinders effective management of epidemics^30^. The diversity of research activities within universities necessitates a mechanism for oversight and integration of supervisory and management efforts. Current research systems could benefit from a more unified approach to enhance decision-making and execution. For example, Pajooheshyar, Pajoohan, Simap, and other research information management systems in the country can be placed under a big research collection and have a managerial, decision-making and implementation approach. Majdkanlo and Javadzadeh asserted that design of information systems of organizations with a systematic approach leads to the integrated production of information in the organization which cover the main functions of the organization. In this approach, the organization is considered as whole, and each of the subsystems corresponds to the appropriate processes of the main functions of the organization^31^. In this regard, the results of the present study are consistent with the study of Mashhadi and et al., they highlighted the need for organizational interactions and intra-organizational collaboration through networking^32^. Kopaei remarked on the importance of collaboration between universities and executive bodies, emphasizing that one of the overarching goals in the Iran’s second development program was to use research as a tool for addressing national challenges^33^. The most significant challenge facing research in the country remains the gap between researchers and policymakers. Namdarian, Abdullahi, and Loncarevic identified barriers to communication between researchers and policymakers, including the inapplicability of research to policy, ineffective connections between policymakers and researchers, resource shortages, and lack of capacity to conduct research related to politics, culture and existing problems in disseminating research finding^34–36^. Karimian and SalehSedghpour also points out that managerial vision, human relations, attitudes, and research capabilities influence research activities. Also mentioned that the barriers in the faculties are affected more than anything by the managerial vision, and therefore, overcoming these barriers depends more on management perspectives, attitudes and methods rather than being affected by availability of financial resources and facilities^37^. The findings of Vătămănescu and et al., research also provide evidence that demonstrates the positive impact of communication between leaders and organizational members. Additionally, competent managers with effective communication skills foster internal communication and dialogue within the organization, increase trust, and enhance team spirit^38^. In numerous studies, which are aligned with the results of the present study, team-building in organizations is emphasized as it leads to a better workplace environment. An organization that encourages team-working indirectly facilitates effective collaboration among all team members, increases job satisfaction, improves productivity, and fosters a more positive and confident workforce^39–41^. It appears that the most significant barriers are related to scientific dimensions, human relationships, and attitudes. To reduce the negative consequences of managerial stability, there are solutions such as: selecting professional managers through merit-based selection, ensuring responsibility and accountability through the establishment of transparency in information and managerial performance, recognizing public oversight, implementing an evaluation system on performance and using the finding to retain managers. Supportive managers can be highly beneficial to an organization in various circumstances. These leaders can increase employee satisfaction and motivation. The evidence showed that in Iran, there is a stronger emphasis on relationships rather than regulatoryism. The performance of governmental organizations, including scientific and research institutions, depends on the opinions of individuals rather than laws. In such an environment, and lack of institutionalized research activities, there is a growing tendency to rely on power and influential individuals rather than principled laws and established principles, and conditions written in books and legal approvals^42^. Based on the results of this study and studies on inflation in recent years, the current budget and resources allocated to research and technology are insufficient to meet the needs for serious and professional work aimed at addressing societal issues and challenges. More attention may need to be directed towards this sector. In Karimian and SalehSedghpour study, financial constraints and the lack of sufficient authority to research centers and researchers in making financial and executive decisions were identified as the major challenges from the researchers’ perspective^37^. Additionally, many studies emphasize that funding should not be spent without a clear purpose. Despite the limited available funds, careful and targeted spending should receive serious and special attention^43–45^. It is essential to ensure that funding is allocated purposefully rather than arbitrarily, as well as to develop policies for financing the research system and allocating public and private resources. Strengthening the role of the private sector in financing and delivering health services is vital for fostering effective collaboration and support in research endeavors.

### Recommendations and conclusion

This study aimed to identify the factors influencing the implementation of research findings in the field of non-communicable diseases. The results indicated that the research system requires strategic direction towards overarching policies, strengthening existing policies, and comprehensive planning. Research managers and policymakers should adopt a critical perspective on each issue and take actions to improve the current situation and address potential challenges. Notably, each of the identified influencing factors has the potential for independent impact on the implementation process. Health system managers and policymakers can utilize the finding of this study, which are part of the recommendations of deputy head of research and technology in medical sciences universities and managers of research development and evaluation, in their future planning and policymaking to enhance a problem-oriented approach to research and to leverage implementation science. Overall, alongside addressing challenges, the research system should also emphasize its facilitative role and adopt effective strategies to optimize and purposefully utilize research capacity for practical actions.

### Limitation

The covid-19 created limitations that made the interviews lengthy and time-consuming. To overcome this problem, Google Meet, Skype, and internal messaging networks were used. The unwillingness of some participants to cooperate in the study, so that, by providing explanations of the importance of the research, partially resolved ambiguities. In qualitative research, claims of generalizability are less evident, and this is one of the main limitations of these studies. For this reason, the researcher must know the type and method of generalizability in qualitative research.

## Data Availability

The data that support the findings of this study are available upon reasonable request by contacting the author(venovel14@gmail.com).
